# Trends in cannabis use disorder and treatment by race and ethnicity, 2002–2019

**DOI:** 10.3389/fpsyt.2025.1689719

**Published:** 2025-11-05

**Authors:** Mireia Triguero Roura, Aabha Vora, Evan L. Eschliman, Pia M. Mauro

**Affiliations:** ^1^ Department of Epidemiology, Columbia University Mailman School of Public Health, New York, NY, United States; ^2^ Center for Pharmacoepidemiology and Treatment Science, Rutgers University, New Brunswick, NJ, United States; ^3^ Department of Biostatistics and Epidemiology, Rutgers School of Public Health, Piscataway, NJ, United States

**Keywords:** cannabis use disorder, cannabis treatment, cannabis treatment need, racialized differences, cannabis trends

## Abstract

**Aims/background:**

As cannabis use continues to increase in the United States, racialized stereotypes and structural barriers to care might lead to inequitable treatment uptake across racialized groups. A greater understanding of racial disparities in cannabis treatment uptake and perceived treatment need among people with cannabis use disorder (CUD) in community-based samples is needed.

**Methods:**

Nationally representative data from the 2002–2019 National Survey on Drug Use and Health (N=1,005,421) included community-based people ages 12+ First, we assessed trends in CUD (i.e., meeting 2+ DSM-5 proxy CUD criteria) by racialized group. Among people with CUD (n=48,768), weighted logistic models regressed any CUD treatment, specialty CUD treatment, and perceived need for CUD treatment on racialized group and year, adjusting for age, gender, education, insurance, and criminal legal system exposure. We use interaction terms to examine group-specific trend differences.

**Results:**

Overall, 2.64% of the US population ages 12+ had CUD, including 2.47% of white, 1.23% of AAPI (Asian American and Pacific Islander), 4.83% of AIAN (American Indian and Alaska Native), 3.70% of Black, and 2.71% of Hispanic people. CUD increased slightly across all groups (overall annual OR: 1.01, 95% CI =1.01,1.02), with AAPI, Hispanic and people with more than one race seeing steeper increases (ORs: 1.02-1.04). Treatment use and perceived need decreased consistently and there were no group differences in these trends across racialized groups with CUD (ORs: 0.93-0.97). Black people with CUD had 21% lower odds of any treatment than white people (aOR: 0.79, 95% CI=0.65, 0.95). All racialized groups (except AAPI people) had higher odds of perceived need than white people (aORs: 1.59–1.73).

**Conclusion:**

Although all racialized groups had increasing CUD and decreasing CUD treatment use during the study period, the observed racialized disparities also persisted. For example, despite a higher prevalence of CUD among Black people compared to white people, Black people with CUD were less likely than their white counterparts to receive treatment and more likely to report perceived need. These disparities in CUD, treatment use, and perceived need underscore the need for CUD-related services overall and tailored services for racialized minorities, and especially Black people.

## 1 Introduction

The landscape of cannabis use in the United States has undergone a significant transformation in recent years, marked by legalization across many states (1), increasing availability, and shifting public attitudes (2). As a result of this expanded access and normalization (3–5), cannabis use has had an upward trend across populations in the US over the last couple decades (6, 7). Heavy cannabis use has been linked to adverse health outcomes, such as respiratory diseases (8) or increased risk of vehicle accidents (9) and can lead to cannabis use disorder (CUD), which is associated with cognitive impairments, psychiatric comorbidities, and functional disruptions across multiple domains of life (10–12). However, research on CUD trends for the same period is mixed. While some studies have shown that CUD has increased among some populations, such as veterans and those with chronic pain (13, 14), research looking at the US population overall and using the DSM-IV criteria shows no increases in CUD, despite some specific criteria trending upward (15).

At the same time, CUD treatment has been declining in recent years (16–18). Treatment for CUD can take various forms; evidence-based medical and specialty treatments for CUD include psychosocial approaches (particularly cognitive-behavioral therapy) proven to be the most effective (19). However, research shows that only a small proportion of adults with CUD receive any form of treatment, and even fewer access specialty care within the healthcare system (16). Individuals with CUD often report receiving support outside of formal medical settings, such as through community-based resources (e.g., churches) or peer support groups not specific to cannabis use, like Alcoholics Anonymous or Narcotics Anonymous (16, 20). In light of low treatment use, an important indicator to measure unmet need for treatment is perceived need for treatment, which has been shown to be a strong predictor of future treatment engagement (21, 22). While findings looking at the general population show that overall utilization of CUD treatment and perceived need has been declining in recent years (16–18), more information on racialized disparities in trends is needed.

Racialized minorities have historically experienced inequities in access to and engagement in substance use disorder treatment (23–25). Structural racism may differentially shape the treatment pathways available to different racialized groups—even when rates of treatment might look nominally similar. Structural barriers, such as discrimination within healthcare systems, culturally unresponsive care, stigma, and limited access to affordable, high-quality services (26, 27), may all contribute to lower rates of treatment uptake among racialized groups. Racialized stereotypes about drug use and who is “deserving” of care may further compound these barriers and shape individuals’ experiences of help-seeking (28–30). The criminal legal system also plays a critical role in shaping racialized disparities in substance use treatment, especially CUD treatment. Racist policies, over-policing of low-income racialized minority communities, and differential criminalization of non-White behaviors (31) have long disproportionately harmed Black and Hispanic populations, leading to large disparities in cannabis-related criminalization (31, 32). At the same time, the criminal legal system is the most common referral source for CUD treatment (33). As a result, Black individuals are overrepresented among those referred to CUD treatment by the courts (33), which may shape not only their access to care but the type and quality of care received because court-mandated treatment can function as both a form of correctional supervision and coerced health intervention. Accounting for exposure to the criminal legal system is necessary to fully understand racial disparities in CUD treatment access and engagement.

Although some research has examined racial differences in cannabis use and CUD prevalence (34), less is known about racial disparities in CUD treatment uptake and perceived need within community-based samples during the era of expanding cannabis legalization. This study aims to (1) describe trends over time in CUD using the DSM-5 proxy measure overall and across racialized groups, and (2) investigate trends in CUD treatment uptake and perceived treatment need overall and among different racialized groups in the United States utilizing nationally representative data from the 2002-2019. Using a proxy of the DSM-5 improves the measurement of CUD by replacing the separate DSM-IV categories of “abuse” and “dependence” with a single construct. The DSM-5 measures removed the potentially racially biased legal criterion and added other clinically relevant symptoms (15, 35, 36). By examining these trends, this research seeks to provide valuable insights into the evolving landscape of CUD and CUD treatment disparities across time and inform the development of more equitable and culturally responsive approaches to care. Throughout the manuscript, “racialized groups” is used instead of racial groups to emphasize the actively constructed nature of “race” and racial groups. “Racialized minorities” refers to the non-dominant racialized groups (i.e. non-White racialized groups). See Hochman (37) for more on this conceptual distinction.

## 2 Methods

### 2.1 Data and sample

*Data sources:* The National Survey on Drug Use and Health (NSDUH) is a nationally representative survey of non-institutionalized individuals aged 12 and above from all 50 states and DC. Topics covered include substance use, disorder, treatment, mental illness, and mental health care. We used public-use NSDUH datasets from 2002-2019, which contained about 56,000 records each year (N = 1,005,421). NSDUH underwent methodological changes that impacted the 2015 data collection and onwards. Variables from 2002–2014 and from 2015–2019 were harmonized where appropriate. We cannot include more recent data because of substantive methodological changes in NSDUH starting in 2021, and the COVID-19 data collection disruptions in 2020 (38–40).

*Sample:* For analyses describing trends in DSM-5 proxy CUD, our sample included all individuals in the NSDUH 2002-2019 (N = 1,005,421). For analyses assessing differences in CUD treatment uptake and perceived need, we restricted our sample to individuals who met past-year DSM-5 proxy CUD (N = 48,768), as described below.

### 2.2 Measures

#### 2.2.1 Outcome measures

*CUD DSM-5-proxy:* Study participants were considered to have DSM-5 proxy CUD if they had two or more of the nine criteria collected in the NSDUH, as described in Compton et al., 15. This measure includes nine of the 11 DSM-5 criteria. DSM-5 CUD criterion 4 (“craving”) and criterion 11 (“withdrawal”) were not assessed in NSDUH before 2020 and were therefore not captured.

*CUD treatment:* Participants were considered as having any past-year CUD treatment if they answered that they had received treatment in any location for any illicit drug use in the past year, and if their last/current treatment episode was for cannabis, as done in previous research (16). Participants were considered as having past-year specialty CUD treatment if they answered that they had received treatment for any illicit drug use in the past year, if their last/current treatment episode was for cannabis, and if this treatment occurred at a specialty facility (i.e., inpatient or outpatient treatment at a hospital, rehabilitation facility, or mental health center).

*Perceived need for CUD treatment:* Perceived need for treatment was measured by asking participants who did not receive any treatment whether they thought they needed counseling or treatment, and participants who did receive treatment whether they thought they needed additional treatment, for their use of “marijuana or hashish” in the past 12 months.

#### 2.2.2 Covariates

*Race/Ethnicity:* We categorized race/ethnicity into the following six mutually exclusive racialized groups: non-Hispanic white, non-Hispanic AAPI (Asian American/Pacific Islander/Native Hawaiian), non-Hispanic AIAN (American Indian and Alaska Native), non-Hispanic Black (Black/African American), Hispanic, and non-Hispanic more than one race.

*Time:* Year was treated as a continuous predictor starting at 0 (for year 2002). Although there was also a major redesign in 2015, previous research has shown that the redesign did not have an effect in the measurement of CUD treatment uptake and perceived treatment need (16).

*Individual control variables:* The following covariates known to be associated with the outcome (16) were included to improve the precision of our estimates and to facilitate comparisons across our main predictors of interest while holding other factors constant. These adjustments are not intended as a confounding control strategy, and the resulting estimates should not be interpreted causally. Participants were categorized as having lifetime CLS exposure if they answered that they had ever been arrested and/or booked. We adjusted for additional sociodemographic variables such as age (12-17, 18-21, 22-25, 26-34, 35+), education (high school or less, some college or college graduate), interviewer-recorded gender (male, female), and health insurance status (private insurance only, public insurance only, both private and public insurance, other, uninsured). NSDUH has been performing imputation using the predictive mean neighborhood (PMN) method since 1999 for several variables in the survey. NSDUH imputes responses for set groups of demographic and drug usage variables, described in the NSDUH 2019 Editing and Imputation Report (41). NSDUH implements this method in three steps: response propensity (determining probability of response) adjustment, prediction modeling (calculating predicted means), and hot-deck imputation (choosing a “donor” whose non-missing data will be used to fill in the missing values in the “recipient’s” data). Education and insurance status were the only variables in our analysis for which imputation procedures were performed. Among our sample of people meeting criteria for DSM-5 Proxy CUD (N = 48,768), N = 8 had imputed values for education, and N = 315 had imputed or logically assigned values for insurance status.

### 2.3 Analytical strategy

We first visually assessed trends in DSM-5 proxy CUD and CUD treatment over time overall and separately for racialized groups with plots fitted and smoothed using loess regression.

To assess trends in CUD over time we ran weighted, unadjusted logistic models regressing DSM-5 proxy CUD on the continuous year variable. To see if trends varied by racialized group we added an interaction term between race and continuous year.

To assess racial disparities in CUD treatment, we ran separate weighted logistic regressions modeling the odds of each outcome (i.e., any treatment, specialty treatment, and perceived treatment need) regressed on race and adjusting for age, education, gender, insurance status, CLS exposure, and continuous year.

All analyses used NSDUH’s complex sampling design and survey weights, which produces nationally representative estimates. All data analyses were performed in STATA version 19.5, and all loess plots were generated in R version 4.4.1 using the ggplot2 package version 3.5.1.

## 3 Results

### 3.1 Descriptive characteristics of sample

**Table 1** explores weighted summary statistics of those meeting criteria for DSM-5 proxy CUD in NSDUH 2002-2019 (N = 48,768). Our weighted sample was 61.8% white, 2.4% AAPI, 1.0% AIAN, 16.7% Black, 15.4% Hispanic, and 2.8% more than one racialized group. The sample was 66.1% male, 27.6% were between the ages 18-21, 44.6% had up to a high school degree, 48.4% had private health insurance, and 43.0% had any lifetime CLS involvement.

**Table 1:** Sample characteristics of people aged 12 and older meeting criteria for past-year DSM-5 proxy CUD, NSDUH 2002-2019 (N = 48,768).

	Overall	White	AAPI	AIAN	Black	Hispanic	More than one
Age							
12-17	13758 (15.8)	8162 (15.6)	259 (13.9)	393 (18.8)	1462 (10.8)	2644 (21.4)	838 (19.0)
18-21	16664 (27.6)	10102 (27.6)	387 (30.9)	390 (26.7)	2310 (24.1)	2612 (30.6)	863 (29.5)
22-25	10878 (18.3)	6440 (17.9)	281 (22.7)	266 (15.8)	1828 (19.1)	1564 (18.9)	499 (15.1)
26-34	4434 (19.9)	2542 (18.8)	88 (18.5)	137 (18.3)	897 (25.4)	559 (18.9)	211 (18.7)
35+	3034 (18.4)	1955 (20.0)	56 (14.0)	115 (20.4)	528 (20.6)	244 (10.1)	136 (17.8)
Education							
High school or less	20425 (44.6)	11522 (41.4)	326 (24.7)	697 (57.6)	3689 (56.5)	3191 (47.6)	1000 (41.4)
Some college or college graduate	14585 (39.6)	9517 (43.1)	486 (61.4)	211 (23.7)	1874 (32.7)	1788 (30.9)	709 (39.7)
12–17 years old	13758 (15.8)	8162 (15.6)	259 (13.9)	393 (18.8)	1462 (10.8)	2644 (21.4)	838 (19.0)
Gender							
Male	30222 (66.1)	18250 (66.2)	693 (67.9)	768 (60.9)	4394 (65.6)	4664 (67.5)	1453 (59.6)
Female	18546 (33.9)	10951 (33.8)	378 (32.1)	533 (39.1)	2631 (34.4)	2959 (32.5)	1094 (40.4)
Health Insurance							
Private	23720 (48.4)	16788 (55.3)	610 (60.0)	206 (17.2)	2186 (32.0)	2864 (39.2)	1066 (43.5)
Public	11683 (22.1)	5219 (17.4)	193 (14.6)	530 (38.5)	2610 (33.5)	2332 (27.7)	799 (29.7)
Public and private	1419 (2.7)	736 (2.5)	34 (2.4)	55 (3.0)	261 (3.2)	230 (2.8)	103 (4.5)
Other	1682 (3.1)	725 (2.7)	39 (4.0)	301 (21.0)	267 (3.7)	217 (2.5)	133 (3.9)
Uninsured	10264 (23.7)	5733 (22.1)	195 (19.0)	209 (20.3)	1701 (27.6)	1980 (27.8)	446 (18.4)
CLS Exposure	19391 (42.9)	11187 (41.7)	286 (25.7)	703 (54.4)	3261 (50.6)	2954 (42.1)	1000 (41.2)

AAPI, American Asian and Pacific Islander; AIAN, American Indian and Alaskan Native; CLS, Criminal legal system; CUD, Cannabis Use Disorder.

### 3.2 Trends in DSM-5 Proxy CUD

**Figure 1:**
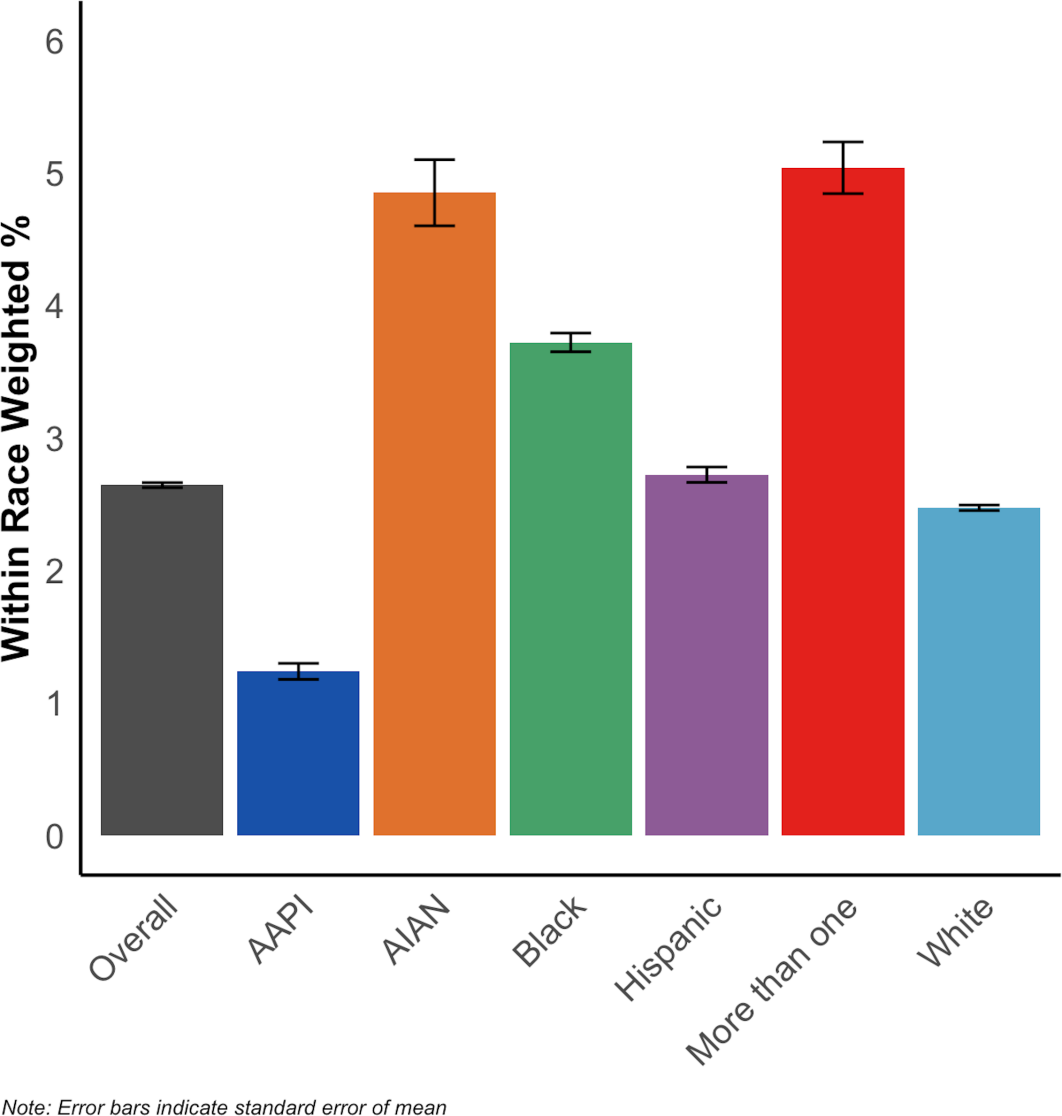
Prevalence of DSM-5 proxy CUD among people aged 12 and older, overall and by racialized group, NSDUH 2002-2019 (N = 1,005,421).

**Figure 1** shows weighted prevalences of DSM-5 proxy CUD overall and across racialized groups among the entire NSDUH 2002–2019 sample (N = 1,005,421). During the study period, 2.6% of the US population aged 12+ met the criteria for DSM-5 proxy CUD. By racialized group, 2.5% of white people, 1.2% of AAPI people, 4.8% of AIAN people, 3.7% of Black people, 2.7% of Hispanic people, and 5.0% of people categorized as more than one racialized group met criteria for DSM-5 proxy CUD.

**Figure 2:**
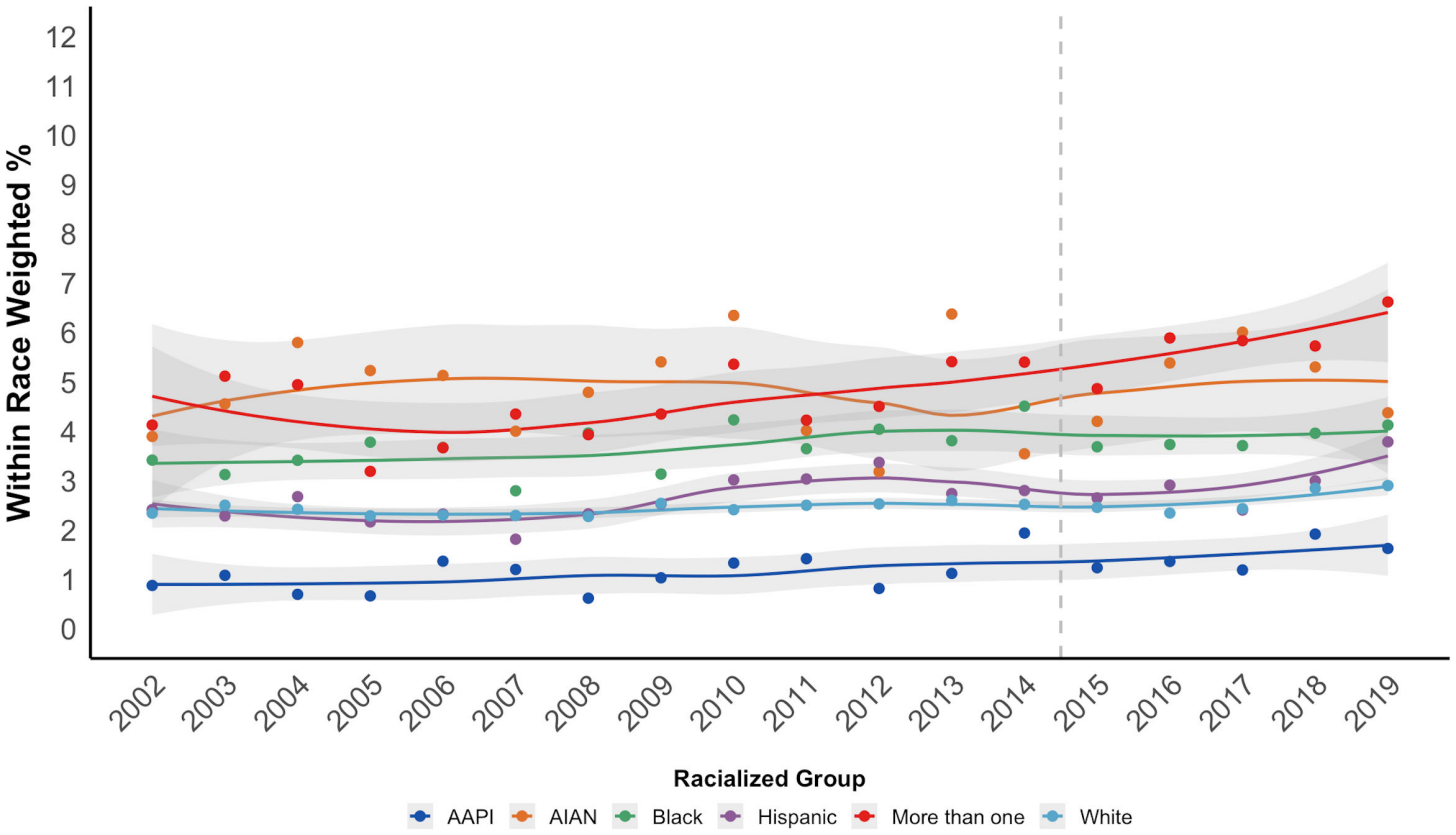
Time trends in prevalence of past-year DSM-5 proxy CUD among people aged 12 and older, by racialized group, NSDUH 2002-2019 (N = 1,005,421).

The overall prevalence of DSM-5 proxy CUD increased from 2.4% in 2002 to 3.2% in 2019 (N = 1,005,421). The odds of meeting criteria for DSM-5 proxy CUD among the US population increased by 1 percentage point per each one-year increase between 2002 and 2019 (annual OR = 1.01, 95% CI = 1.01, 1.02). **Figure 2** shows the prevalence of DSM-5 proxy CUD increased across all racialized groups. Weighted logistic models regressing CUD on continuous year in **Table 2** show the increase in odds of meeting criteria for DSM-5 proxy CUD per each 1 year increase were higher for AAPI people (interaction OR = 1.03, 95% CI = 1.01, 1.05), Hispanic people (interaction OR = 1.01, 95% CI = 1.00, 1.02), and people of more than one race (interaction OR = 1.02, 95% CI = 1.00, 1.04), compared to white people. There were no significant trend differences comparing Black and AIAN people to white people.

**Table 2:** Yearly trends in prevalence of DSM-5 proxy cannabis use disorder (CUD) among people aged 12 and older with interaction by racialized group, NSDUH 2002-2019 (N = 1,005,421).

	DSM-5 proxy CUD
	aOR (95% CI)
Year	
Per 1 year increase	**1.01 (1.01, 1.01)**
Race	
White	Reference
AAPI	**0.35 (0.28, 0.43)**
AIAN	**2.12 (1.67, 2.69)**
Black	**1.47 (1.34, 1.61)**
Hispanic	0.96 (0.87, 1.06)
More than one	**1.66 (1.36, 2.02)**
Race × 1 year increase	
White*1 year increase	Reference
AAPI*1 year increase	**1.03 (1.01, 1.05)**
AIAN*1 year increase	0.99 (0.97, 1.01)
Black*1 year increase	1.00 (1.00, 1.01)
Hispanic*1 year increase	**1.01 (1.00, 1.02)**
More than one*1 year increase	**1.02 (1.00, 1.04)**

CUD, Cannabis Use Disorder.

Bolded values indicate p < 0.05.

### 3.3 Trends in CUD treatment uptake and unmet need

**Figure 3:**
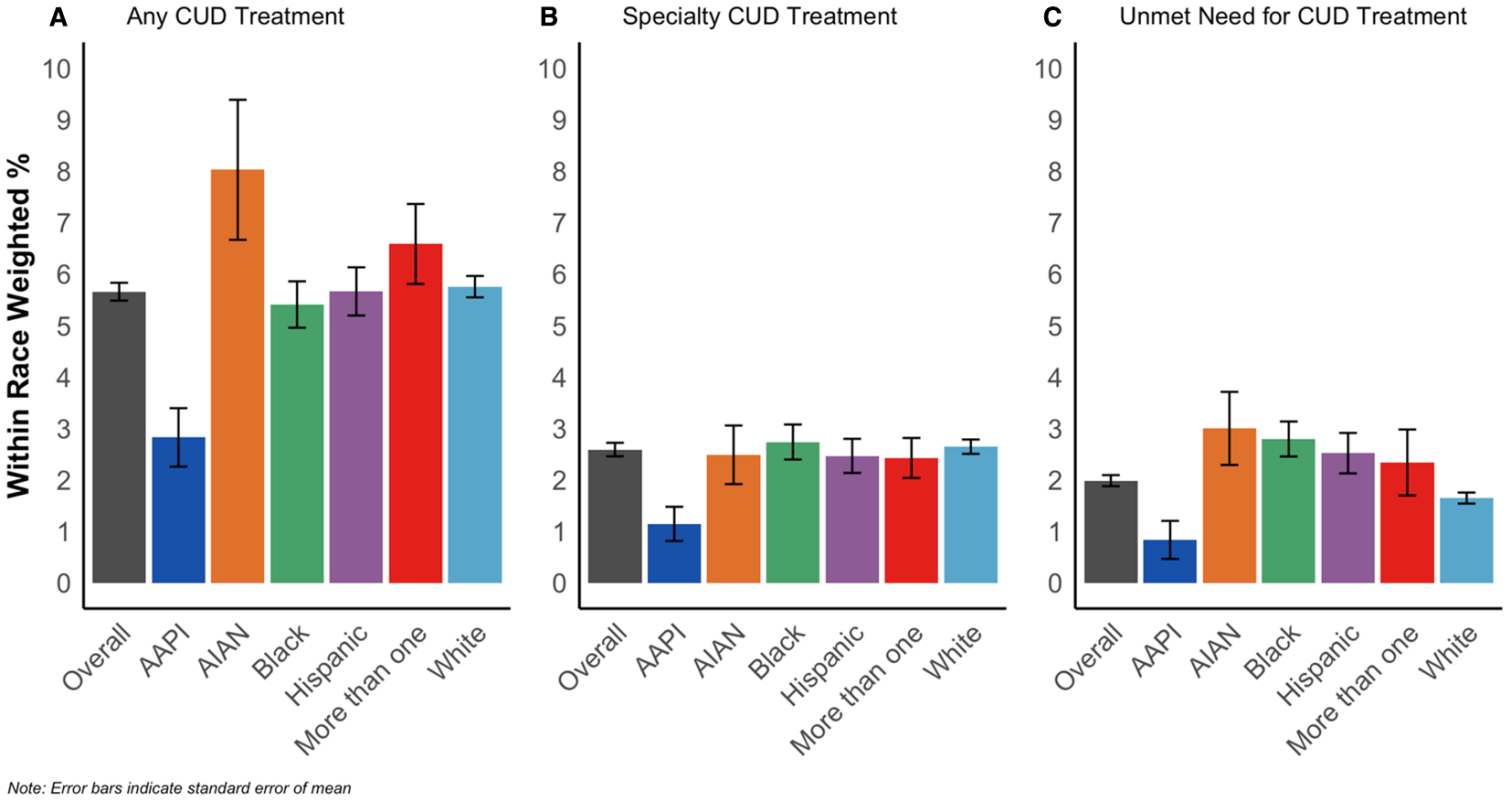
Prevalence of any CUD treatment **(A)**, specialty CUD treatment **(B)**, and unmet CUD treatment need **(C)** among people with DSM-5 proxy CUD, overall and by racialized group, NSDUH 2002-2019 (N = 48,768).

**Figure 3** shows the prevalence of CUD treatment and perceived need by racialized groups among those with DSM-5 proxy CUD (N = 48,768). 5.8% of white people, 2.8% of AAPI people, 8.0% of AIAN people, 5.4% of Black people, 5.7% of Hispanic people, and 6.6% of people categorized as more than one race received any CUD treatment, while 2.6% of white people, 1.1% of AAPI people, 2.5% of AIAN people, 2.7% of Black people, 2.5% of Hispanic people, and 2.4% of people categorized as more than one race received specialty treatment. Finally, 1.7% of white people, 0.8% of AAPI people, 3.0% of AIAN people, 2.8% of Black people, 2.5% of Hispanic people, and 2.3% of people of more than one race reported unmet need for CUD treatment.

**Figure 4:**
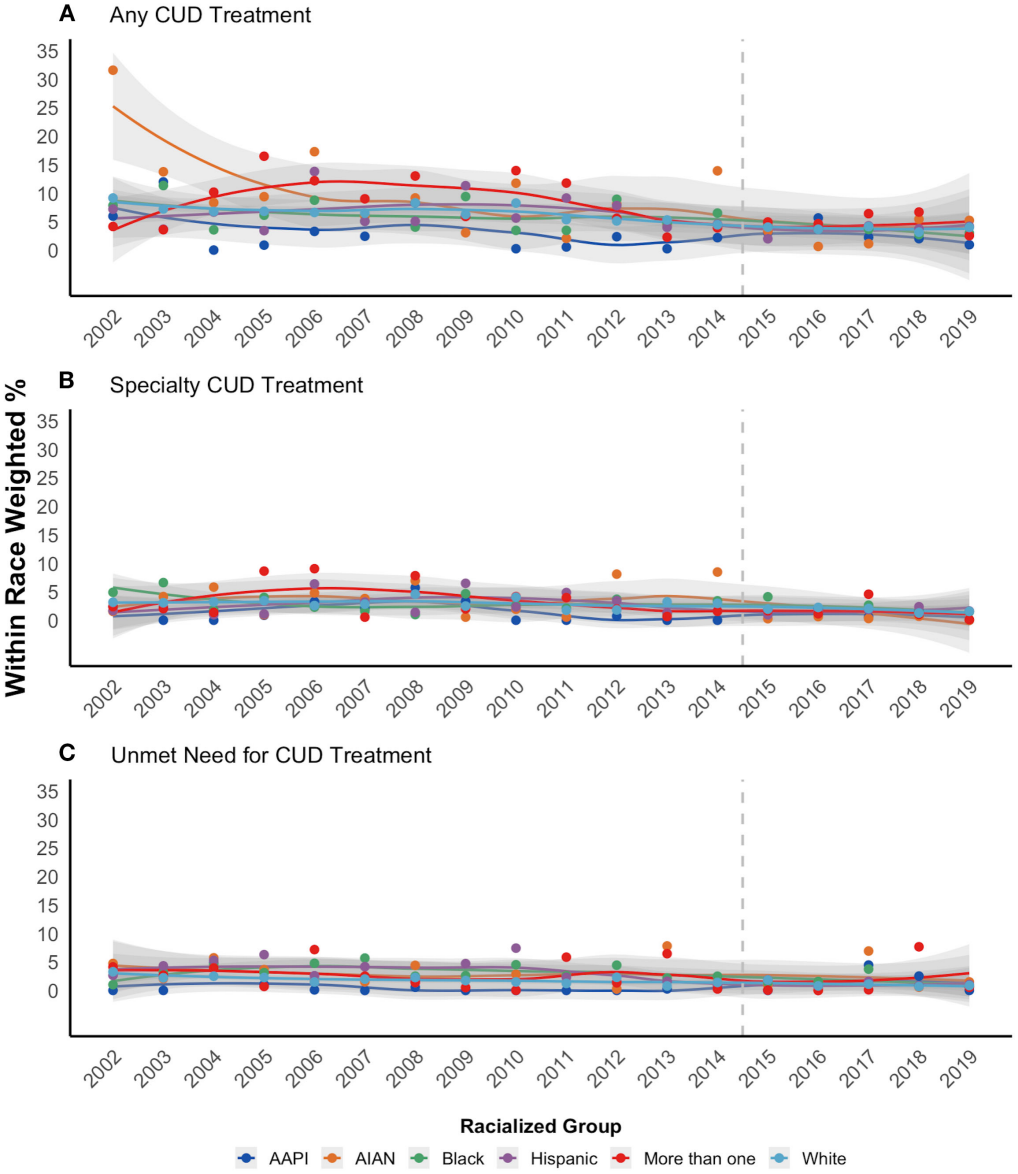
Time trends in prevalence of past-year CUD treatment **(A)**, specialty CUD treatment **(B)**, and unmet CUD treatment need **(C)** among people aged 12 and older who meet criteria for DSM-5 proxy CUD, by racialized group, NSDUH 2002-2019 (N = 48,768).

**Figure 4** shows that all three CUD treatment outcomes show a downward trend across racialized groups. In **Table 3** we report logistic model estimates confirming this downward trend. For every one-year increase, the odds of reporting CUD treatment decreased by 4% (OR: 0.96, 95% CI = 0.95, 0.98) for any treatment and by 3% for specialty treatment (aOR: 0.97, 95% CI = 0.95, 0.99). For perceived need, the odds decreased by 7% (aOR: 0.93, 95% CI = 0.90, 0.95) for every one-year increase. Testing interaction between continuous year and race found no significant differences in the downward treatment trend across racialized groups (**Supplementary Table 1**).

**Table 3:** Fully adjusted logistic regression models assessing relationship between treatment outcomes and race among people meeting criteria for DSM-5 Proxy CUD, NSDUH 2002-2019 (N = 48,768).

	Any CUD treatment	Specialty CUD treatment	Perceived CUD treatment need
	aOR (95% CI)	aOR (95% CI)	aOR (95% CI)
Year			
Per 1 year increase	**0.96 (0.95, 0.98)**	**0.97 (0.95, 0.99)**	**0.93 (0.91, 0.95)**
Race			
White	Reference	Reference	Reference
AAPI	0.68 (0.45, 1.04)	0.64 (0.36, 1.15)	0.63 (0.26, 1.55)
AIAN	0.91 (0.62, 1.33)	**0.57 (0.34, 0.93)**	**1.72 (1.03, 2.90)**
Black	**0.79 (0.65, 0.95)**	0.80 (0.60, 1.05)	**1.59 (1.21, 2.09)**
Hispanic	0.89 (0.74, 1.07)	0.81 (0.62, 1.07)	**1.73 (1.20, 2.48)**
More than one	1.11 (0.85, 1.46)	0.82 (0.58, 1.18)	1.59 (0.91, 2.81)
Age			
12-17	Reference	Reference	Reference
18-21	**0.55 (0.48, 0.63)**	**0.80 (0.66, 0.96)**	0.84 (0.66, 1.06)
22-25	**0.49 (0.41, 0.58)**	**0.77 (0.60, 0.99)**	0.83 (0.64, 1.07)
26-34	**0.48 (0.38, 0.62)**	0.82 (0.59, 1.13)	1.31 (0.92, 1.86)
35+	**0.54 (0.43, 0.69)**	0.76 (0.54, 1.07)	**1.60 (1.03, 2.47)**
Education			
High school or less	Reference	Reference	Reference
Some college or college graduate	**0.68 (0.57, 0.82)**	**0.56 (0.44, 0.71)**	1.11 (0.87, 1.41)
Gender			
Male	Reference	Reference	Reference
Female	1.07 (0.95, 1.21)	**1.25 (1.06, 1.49)**	1.21 (0.97, 1.51)
Insurance			
Private only	Reference	Reference	Reference
Public only	**1.49 (1.27, 1.75)**	**1.93 (1.51, 2.46)**	**1.35 (1.01, 1.80)**
Public and private	1.23 (0.89, 1.71)	**1.90 (1.19, 3.04)**	0.67 (0.36, 1.23)
Other	**1.90 (1.29, 2.80)**	1.58 (0.94, 2.66)	0.91 (0.49, 1.70)
Uninsured	1.09 (0.91, 1.31)	1.09 (0.84, 1.41)	1.19 (0.90, 1.56)
CLS Exposure			
No	Reference	Reference	Reference
Yes	**4.01 (3.46, 4.65)**	**4.37 (3.47, 5.48)**	**1.41 (1.12, 1.79)**

AAPI, American Asian and Pacific Islander; AIAN, American Indian and Alaskan Native; CLS, Criminal legal system; CUD, Cannabis Use Disorder.

Bolded values indicate p < 0.05.

### 3.4 Racial differences in CUD treatment uptake and unmet need

**Table 3** displays weighted logistic model results regressing our three outcomes on racialized group accounting for continuous year, age group, education, binary gender, health insurance status, and lifetime CLS exposure. The adjusted odds of any CUD treatment were significantly lower among Black people compared to white people (aOR = 0.79, 95% CI = 0.65, 0.95), but not for other racialized groups. AIAN people had significantly lower odds of specialty CUD treatment compared to white people (aOR = 0.57, 95% CI = 0.34, 0.93), but this relationship was not significant for other racialized groups. Black (aOR = 1.59, 95% CI = 1.21, 2.09), Hispanic (aOR = 1.73, 95% CI = 1.20, 2.49), and AIAN (aOR = 1.72, 95% CI = 1.03, 2.90) people all had higher odds of perceived treatment need compared to white participants. The odds of any CUD treatment (aOR = 4.01, 95% CI = 3.46, 4.65), specialty CUD treatment (aOR = 4.37, 95% CI = 3.47, 5.48), and perceived need for CUD treatment (aOR = 1.41, 95% CI = 1.12, 1.79) were all higher for those with lifetime CLS exposure compared to those without.

## 4 Discussion

This study assessed trends in CUD as well as CUD treatment use and perceived need for treatment in 2002–2019 by racial group in a nationally representative sample, describing racial disparities in treatment access and need as well as changes over time. DSM-5 proxy CUD prevalences increased across the population, signaling a growing treatment need in the US. In this same time period, both specialty and any CUD treatment uptake and perceived treatment need diminished over time for all racialized groups with past-year CUD. Decreases in CUD treatment were consistent across racialized groups, which indicates that these inequities have remained stagnant over time.

All racialized minorities with DSM-5 proxy CUD (with the exception of AAPI people) were more likely to report unmet perceived need for treatment when compared to white people with DSM-5 proxy CUD. Looking specifically at treatment, Black people with DSM-5 proxy CUD were less likely than white people to report receiving any CUD treatment, in spite of Black people having slightly higher rates of DSM-5 proxy CUD than white people among the general population. As for specialty treatment, AIAN people with DSM-5 proxy CUD were much less likely to receive specialty treatment than white people with DSM-5 proxy CUD. Decreases in treatment across groups are consistent with previous findings of CUD treatment decreases by age and among young adults (16, 18), and signal that CUD treatment gaps are growing across a range of demographic groups.

There are several reasons that could explain this overall diminishing trend in treatment while CUD increased. Increased access and shifts in public opinion in the last couple decades could have contributed to higher cannabis normalization (3–5), whereas people with CUD might be less likely to perceive a need for treatment and therefore seek out help. However, general barriers to access (such as insurance limiting access to treatment and brief interventions) could also be driving some of this diminishing trends. Changes in the legal status of cannabis across the country, particularly in states with cannabis dispensaries, might have also contributed to the overall observed reduction in treatment uptake (17). Since 2010 arrests for cannabis possession have been diminishing across the US (42), and research has observed that court-mandated treatment episodes in public hospitals have also experienced a downward trend during the same period (43). Another possibility is that the reduction in the prevalence of treatment uptake is a direct result of the increase in CUD prevalence among the general population: if increased access and normalization of CUD are contribution to increases in CUD prevalences but treatment system capacity has remained stagnant—because of structural constraints such as limited infrastructure, workforce shortages, or funding—this would create a supply-side bottleneck, producing an apparent decline in the proportion of people with CUD receiving treatment even if demand is rising.

Together, our findings indicate a general pattern of ongoing racialized access to CUD treatment. Racism and other racialized structural factors might explain this persistent gap. Access to medical services in the US is racialized (44). Black and Hispanic people are more likely to live in areas with fewer providers (27, 45, 46), and more likely to be overrepresented in low-paying jobs with less comprehensive health benefits. At the same time, racialized stigma toward cannabis use, as well as different groups’ perceptions of and lived experiences with the medical systems might impede CUD treatment among different racialized groups (26, 30, 32, 47). Importantly, access to treatment for CUD remains extremely low and the vast majority of people with a disorder do not access treatment.

Lastly, it is important to contextualize our understanding of racialized disparities in treatment uptake at the intersection of SUD treatment and the criminal legal system. Court-mandated treatment makes up the biggest share of referrals for CUD treatment episodes (33; TEDS-A, 2000-2023). Black and Hispanic people are over-represented in their CLS exposure, yet our analyses show that Black people with a CUD are less likely to receive treatment (although not specialty treatment) and more likely to report unmet treatment need. This indicates that, despite racialized structures that overly punish Black people’s consumption of cannabis, and a real reported need for treatment, the system is failing to meet this need.

### 4.1 Limitations and future research

The NSDUH does not directly measure our main outcomes of treatment need and uptake. Instead, following Askari et al., 16, we constructed composite measures of past-year CUD treatment based on multiple NSDUH variables. This may have resulted in people who received CUD treatment in the past year being misclassified as not having received CUD treatment if it was not their most recent or current treatment. The DSM-5 proxy measure excludes cravings and withdrawal symptoms, which might be underreporting the number of people with mild CUD (35, 48). Due to a structural redesign in the NSDUH, we could not include data after 2020. Because several covariates (e.g., education, insurance status, CLS exposure) are correlated with race and may shift over time, our models may be subject to collinearity, which could inflate standard errors and make it more difficult to isolate and interpret the independent and interactive effects of race and year. Finally, the NSDUH does not include institutionalized people, which makes our findings limited to non-institutionalized populations. Given the disproportionate rates of incarceration among Black and Hispanic populations, we might be underestimating some of the racialized gaps in CUD and CUD treatment among these groups.

Future research should try to understand how structural and cultural factors might explain why racialized minorities are more likely to report unmet treatment need and less likely to receive treatment for their CUD. Future research and clinical efforts should also address barriers to problem recognition, which could include increasing consistency in screening and brief interventions for cannabis (49). Growing social acceptability of cannabis and limited awareness of CUD symptoms, along with minimization of associated harms might prevent people from accessing treatment. More research is needed in understanding whether these barriers also vary across racialized groups. Finally, future research should investigate how changes in the criminalization of cannabis use and the downward trends in arrests and court-mandated CUD treatment (TEDS-A, 2000-2023) may be contributing to the observed downward trends in treatment uptake and need, as well as how CLS exposure, and specifically what types of exposure (e.g., arrest vs parole), fit in with the racial disparities in treatment presented in this study.

## Data Availability

Publicly available datasets were analyzed in this study. This data can be found here: https://www.samhsa.gov/data/data-we-collect/nsduh-national-survey-drug-use-and-health/national-releases/. Data for 2002-2019 were used.

## Supplementary material

Supplementary Table 1 Fully adjusted logistic regression models assessing interaction between continuous year and race on prevalence of CUD treatment among people meeting criteria for DSM-5 Proxy CUD, NSDUH 2002-2019 (N = 48,768). AAPI, American Asian and Pacific Islander; AIAN, American Indian and Alaskan Native; CLS, Criminal legal system; CUD, Cannabis Use Disorder. Bolded values indicate p < 0.05.

## References

[1] State Medical Cannabis Laws. (2025). Available online at: https://www.ncsl.org/health/state-medical-cannabis-laws.

[2] Levy NS Mauro PM Mauro CM Segura LE Martins SS. Joint perceptions of the risk and availability of Cannabis in the United States 2002-2018. Drug Alcohol Depend. (2021) 226:108873. doi: 10.1016/j.drugalcdep.2021.108873, PMID: 34275699 PMC8478130

[3] Lamy FR Daniulaityte R Sheth A Nahhas RW Martins SS Boyer EW. Those edibles hit hard”: Exploration of Twitter data on cannabis edibles in the U.S. Drug Alcohol Depend. (2016) 164:64–70. doi: 10.1016/j.drugalcdep.2016.04.029, PMID: 27185160 PMC4893972

[4] Martins SS Segura LE Levy NS Mauro PM Mauro CM Philbin MM. Racial and ethnic differences in cannabis use following legalization in US states with medical cannabis laws. JAMA Network Open. (2021) 4:e2127002. doi: 10.1001/jamanetworkopen.2021.27002, PMID: 34570205 PMC8477268

[5] Schuermeyer J Salomonsen-Sautel S Price RK Balan S Thurstone C Min S-J. Temporal trends in marijuana attitudes, availability and use in Colorado compared to non-medical marijuana states: 2003–11. Drug Alcohol Depend. (2014) 140:145–55. doi: 10.1016/j.drugalcdep.2014.04.016, PMID: 24837585 PMC4161452

[6] Han BH Yang KH Cleland CM Palamar JJ. Trends in past-month cannabis use among older adults. JAMA Internal Med. (2025) 185:881–3. doi: 10.1001/jamainternmed.2025.1156, PMID: 40455425 PMC12131166

[7] Hasin D Walsh C. Trends over time in adult cannabis use: A review of recent findings. Curr Opin Psychol. (2021) 38:80–5. doi: 10.1016/j.copsyc.2021.03.005, PMID: 33873044 PMC8905582

[8] Karila L Roux P Rolland B Benyamina A Reynaud M Aubin H-J. Acute and long-term effects of cannabis use: A review. Curr Pharm Design. (2014) 20:4112–8. doi: 10.2174/13816128113199990620, PMID: 24001294

[9] Asbridge M Hayden JA Cartwright JL. Acute cannabis consumption and motor vehicle collision risk: Systematic review of observational studies and meta-analysis. BMJ (Clinical Res Ed.). (2012) 344:e536. doi: 10.1136/bmj.e536, PMID: 22323502 PMC3277079

[10] Broyd SJ van Hell HH Beale C Yücel M Solowij N. Acute and chronic effects of cannabinoids on human cognition—A systematic review. Biol Psychiatry. (2016) 79:557–67. doi: 10.1016/j.biopsych.2015.12.002, PMID: 26858214

[11] Hasin DS Kerridge BT Saha TD Huang B Pickering R Smith SM. Prevalence and correlates of DSM-5 cannabis use disorder 2012-2013: findings from the national epidemiologic survey on alcohol and related conditions–III. Am J Psychiatry. (2016) 173:588–99. doi: 10.1176/appi.ajp.2015.15070907, PMID: 26940807 PMC5026387

[12] Volkow ND Swanson JM Evins AE DeLisi LE Meier MH Gonzalez R. Effects of cannabis use on human behavior, including cognition, motivation, and psychosis: A review. JAMA Psychiatry. (2016) 73:292. doi: 10.1001/jamapsychiatry.2015.3278, PMID: 26842658

[13] Hasin DS Saxon AJ Malte C Olfson M Keyes KM Gradus JL. Trends in cannabis use disorder diagnoses in the U.S. Veterans health administration 2005–2019. Am J Psychiatry. (2022) 179:748–57. doi: 10.1176/appi.ajp.22010034, PMID: 35899381 PMC9529770

[14] Mannes ZL Malte CA Olfson M Wall MM Keyes KM Martins SS. Increasing risk of cannabis use disorder among U.S. veterans with chronic pain: 2005-2019. Pain. (2023) 164:2093–103. doi: 10.1097/j.pain.0000000000002920, PMID: 37159542 PMC10524371

[15] Compton WM Han B Jones CM Blanco C. Cannabis use disorders among adults in the United States during a time of increasing use of cannabis. Drug Alcohol Depend. (2019) 204:107468. doi: 10.1016/j.drugalcdep.2019.05.008, PMID: 31586809 PMC7028308

[16] Askari MS Keyes KM Mauro PM. Cannabis use disorder treatment use and perceived treatment need in the United States: Time trends and age differences between 2002-2019. Drug Alcohol Depend. (2021) 229:109154. doi: 10.1016/j.drugalcdep.2021.109154, PMID: 34741874 PMC8671260

[17] Mauro PM Gutkind S Askari MS Hasin DS Samples H Mauro CM. Associations between cannabis policies and state-level specialty cannabis use disorder treatment in the United States 2004–2019. Drug Alcohol Depend. (2024) 257:111113. doi: 10.1016/j.drugalcdep.2024.111113, PMID: 38382162 PMC11736659

[18] Mennis J Stahler GJ McKeon TP. Young adult cannabis use disorder treatment admissions declined as past month cannabis use increased in the U.S.: An analysis of states by year 2008–2017. Addictive Behav. (2021) 123:107049. doi: 10.1016/j.addbeh.2021.107049, PMID: 34303941

[19] Gates PJ Sabioni P Copeland J Le Foll B Gowing L. Psychosocial interventions for cannabis use disorder. Cochrane Database Systematic Rev. (2016) 2016:CD005336. doi: 10.1002/14651858.CD005336.pub4, PMID: 27149547 PMC4914383

[20] Mauro PM Kaur N Askari MS Keyes KM. Alcohol or drug self-help use among adults in the United States: age, period, and cohort effects between 2002 and 2018. Int J Ment Health Addict. (2024) 22:2667–81. doi: 10.1007/s11469-023-01012-2, PMID: 36785551 PMC9907883

[21] Haughwout SP Harford TC Castle I-JP Grant BF. Treatment utilization among adolescent substance users: findings from the 2002 to 2013 national survey on drug use and health. Alcoholism: Clin Exp Res. (2016) 40:1717–27. doi: 10.1111/acer.13137, PMID: 27427179

[22] Mojtabai R Crum RM. Perceived unmet need for alcohol and drug use treatments and future use of services: Results from a longitudinal study. Drug Alcohol Depend. (2013) 127:59–64. doi: 10.1016/j.drugalcdep.2012.06.012, PMID: 22770461 PMC3488160

[23] Ben J Cormack D Harris R Paradies Y. Racism and health service utilisation: A systematic review and meta-analysis. PloS One. (2017) 12:e0189900. doi: 10.1371/journal.pone.0189900, PMID: 29253855 PMC5734775

[24] Cook BL Alegría M. Racial-ethnic disparities in substance abuse treatment: The role of criminal history and socioeconomic status. Psychiatr Serv (Washington D.C.). (2011) 62:1273–81. doi: 10.1176/ps.62.11.pss6211_1273, PMID: 22211205 PMC3665009

[25] Matsuzaka S Knapp M. Anti-racism and substance use treatment: Addiction does not discriminate, but do we? J Ethnicity Subst Abuse. (2020) 19:567–93. doi: 10.1080/15332640.2018.1548323, PMID: 30642230

[26] Earnshaw VA Mousavi M Qiu X Fox AB. Mental illness and substance use disorder stigma: mapping pathways between structures and individuals to accelerate research and intervention. Annu Rev Clin Psychol. (2025) 21:85–111. doi: 10.1146/annurev-clinpsy-081423-023228, PMID: 39805034

[27] Tadmon D Bearman PS. Differential spatial-social accessibility to mental health care and suicide. Proc Natl Acad Sci. (2023) 120:e2301304120. doi: 10.1073/pnas.2301304120, PMID: 37126686 PMC10175830

[28] Hansen H Roberts SK. Two tiers of biomedicalization: METHADONE buprenorphine and the racial politics of addiction treatment. In: Netherland J, editor. Critical perspectives on addiction (2012). p. 79–102. doi: 10.1108/S1057-6290(2012)0000014008

[29] Hatcher AE Mendoza S Hansen H. At the expense of a life: race, class, and the meaning of buprenorphine in pharmaceuticalized “Care. Subst Use Misuse. (2018) 53:301–10. doi: 10.1080/10826084.2017.1385633, PMID: 29161171 PMC5901978

[30] Walters SM Kerr J Cano M Earnshaw V Link B. Intersectional stigma as a fundamental cause of health disparities: A case study of how drug use stigma intersecting with racism and xenophobia creates health inequities for Black and Hispanic persons who use drugs over time. Stigma Health. (2023) 8:325–43. doi: 10.1037/sah0000426, PMID: 37744082 PMC10516303

[31] Najdowski CJ Stevenson MC. A call to dismantle systemic racism in criminal legal systems. Law Hum Behav. (2022) 46:398–414. doi: 10.1037/lhb0000510, PMID: 36521112

[32] Hammarlund R Crapanzano K Luce L Mulligan L Ward K. Review of the effects of self-stigma and perceived social stigma on the treatment-seeking decisions of individuals with drug- and alcohol-use disorders. Subst Abuse Rehabil. (2018) 9:115–36. doi: 10.2147/SAR.S183256, PMID: 30538599 PMC6260179

[33] McElrath K Taylor A Tran KK. Black-white disparities in criminal justice referrals to drug treatment: addressing treatment need or expanding the diagnostic net? Behav Sci (Basel Switzerland). (2016) 6:21. doi: 10.3390/bs6040021, PMID: 27706092 PMC5197934

[34] Wu L-T Zhu H Swartz MS. Trends in cannabis use disorders among racial/ethnic population groups in the United States. Drug Alcohol Depend. (2016) 165:181–90. doi: 10.1016/j.drugalcdep.2016.06.002, PMID: 27317045 PMC4939114

[35] Compton WM Einstein EB Han B. 12-month prevalence estimates of substance use disorders using DSM-5 versus DSM-IV criteria among U.S. Nonelderly adults with substance use. Am J Psychiatry. (2024) 181:1018–21. doi: 10.1176/appi.ajp.20231060, PMID: 39482948

[36] Fink DS Shmulewitz D Mannes ZL Stohl M Livne O Wall M. Construct validity of DSM-5 cannabis use disorder diagnosis and severity levels in adults with problematic substance use. J Psychiatr Res. (2022) 155:387–94. doi: 10.1016/j.jpsychires.2022.09.016, PMID: 36182768 PMC9590423

[37] Hochman A. Racialization: A defense of the concept. Ethnic Racial Stud. (2019) 42:1245–62. doi: 10.1080/01419870.2018.1527937

[38] 2015 NSDUH questionnaire redesign impact assessment, final report. CBHSQ Data. Available online at: https://www.samhsa.gov/data/report/2015-nsduh-questionnaire-redesign-impact-assessment-final-report.

[39] 2021 national survey on drug use and health: methodological summary and definitions . Available online at: https://www.samhsa.gov/data/sites/default/files/reports/rpt39442/2021NSDUHMethodSummDefs100422/2021NSDUHMethodSummDefs100422.htm.

[40] 2020 national survey on drug use and health: methodological summary and definitions. Available online at: https://www.samhsa.gov/data/sites/default/files/reports/rpt35330/2020NSDUHMethodSummDefs092421/2020NSDUHMethodsSummDefs092421.htm.

[41] Substance Abuse and Mental Health Services Administration. 2019 NSDUH MRB editing and imputation report. U.S. Department of Health and Human Services (2020). Available online at: https://www.samhsa.gov/data/sites/default/files/reports/rpt34660/NSDUHmrbEditImputation2019.pdf.

[42] Crime in the U.S. (2025). FBI. Available online at: https://ucr.fbi.gov/crime-in-the-u.s.

[43] Treatment episode data set—Admissions (TEDS-A) series . Available online at: https://www.icpsr.umich.edu/web/ICPSR/series/56.

[44] Lê Cook B McGuire TG Lock K Zaslavsky AM. Comparing methods of racial and ethnic disparities measurement across different settings of mental health care. Health Serv Res. (2010) 45:825–47. doi: 10.1111/j.1475-6773.2010.01100.x, PMID: 20337739 PMC2875762

[45] Acevedo A Panas L Garnick D Acevedo-Garcia D Miles J Ritter G. Disparities in the treatment of substance use disorders: does where you live matter? J Behav Health Serv Res. (2018) 45:533. doi: 10.1007/s11414-018-9586-y, PMID: 29435862 PMC6087681

[46] Eberth JM Hung P Benavidez GA Probst JC Zahnd WE McNatt M-K. The problem of the color line: spatial access to hospital services for minoritized racial and ethnic groups. Health Affairs. (2022) 41:237–46. doi: 10.1377/hlthaff.2021.01409, PMID: 35130071

[47] Adams C Chatterjee A Harder BM Mathias LH. Beyond unequal access: Acculturation, race, and resistance to pharmaceuticalization in the United States. SSM - Population Health. (2018) 4:350–7. doi: 10.1016/j.ssmph.2018.04.003, PMID: 29854920 PMC5976842

[48] Hasin D Stohl M. Higher prevalence estimates of substance use disorders with DSM-5 versus DSM-IV criteria among U.S. Nonelderly adults with substance use: the role of DSM-IV diagnostic orphans. Am J Psychiatry. (2024) 181:955–7. doi: 10.1176/appi.ajp.20240852, PMID: 39482952

[49] Gette JA Regan T Schumacher JA. Screening, brief intervention, and referral to treatment (SBIRT) for cannabis: A scoping review. J Subst Use Addict Treat. (2023) 146:208957. doi: 10.1016/j.josat.2023.208957, PMID: 36880902

