# Supplementary material for: Trends in cannabis use disorder and treatment by race and ethnicity, 2002–2019
Source: Front Psychiatry. 2025 Nov 5;16:1689719. doi: 10.3389/fpsyt.2025.1689719 (PMC12626980; doi:10.3389/fpsyt.2025.1689719)
Supplement: Supplementary Table 1 — Fully adjusted logistic regression models assessing interaction between continuous year and race on prevalence of CUD treatment among people meeting criteria for DSM-5 Proxy CUD, NSDUH 2002-2019 (N = 48,768). AAPI, American Asian and Pacific Islander; AIAN, American Indian and Alaskan Native; CLS, Criminal legal system; CUD, Cannabis Use Disorder. Bolded values indicate p < 0.05. [file Table1.docx]

Supplemental Table 1: Fully adjusted logistic regression models assessing interaction between continuous year and race on prevalence of CUD treatment among people meeting criteria for DSM-5 Proxy CUD, NSDUH 2002-2019 (N = 48,768)

|  | **Any treatment** | **Specialty treatment** | **Perceived treatment need** |
| --- | --- | --- | --- |
|  | **aOR (95% CI)** | **aOR (95% CI)** | **aOR (95% CI)** |
| **Year** |  |  |  |
| Per 1 year increase | **0.96 (0.95, 0.98)** | **0.97 (0.95, 0.99)** | **0.93 (0.90, 0.95)** |
| **Race** |  |  |  |
| White | Reference | Reference | Reference |
| AAPI | 0.80 (0.30, 2.10) | 0.71 (0.21, 2.43) | 0.08 (0.00, 1.72) |
| AIAN | 1.55 (0.71, 3.38) | 0.79 (0.39, 1.62) | 1.27 (0.50, 3.20) |
| Black | 0.78 (0.54, 1.14) | 0.83 (0.46, 1.48) | 1.33 (0.85, 2.07) |
| Hispanic | 0.76 (0.54, 1.08) | 0.68 (0.41, 1.12) | **2.25 (1.33, 3.79)** |
| More than one | 1.10 (0.66, 1.85) | 1.16 (0.59, 2.31) | 1.11 (0.39, 3.18) |
| **Race*Year** |  |  |  |
| White*Year | Reference | Reference | Reference |
| AAPI*Year | 0.98 (0.90, 1.08) | 0.99 (0.89, 1.11) | 1.19 (0.94, 1.50) |
| AIAN*Year | 0.94 (0.87, 1.01) | 0.96 (0.89, 1.04) | 1.04 (0.95, 1.13) |
| Black*Year | 1.00 (0.96, 1.04) | 1.00 (0.94, 1.05) | 1.02 (0.98, 1.06) |
| Hispanic*Year | 1.02 (0.98, 1.05) | 1.02 (0.97, 1.07) | 0.97 (0.92, 1.02) |
| More than one*Year | 1.00 (0.95, 1.05) | 0.97 (0.91, 1.03) | 1.04 (0.93, 1.16) |
| **Age** |  |  |  |
| 12-17 | Reference | Reference | Reference |
| 18-21 | **0.55 (0.48, 0.63)** | **0.80 (0.66, 0.96)** | 0.84 (0.66, 1.07) |
| 22-25 | **0.49 (0.41, 0.58)** | **0.77 (0.60, 0.99)** | 0.83 (0.64, 1.07) |
| 26-34 | **0.49 (0.38, 0.62)** | 0.82 (0.59, 1.13) | 1.31 (0.92, 1.86) |
| 35+ | **0.54 (0.43, 0.69)** | 0.76 (0.54, 1.07) | **1.61 (1.04, 2.49)** |
| **Education** |  |  |  |
| High school or less | Reference | Reference | Reference |
| Some college or college graduate | **0.68 (0.57, 0.82)** | **0.56 (0.44, 0.71)** | 1.11 (0.87, 1.41) |
| **Gender** |  |  |  |
| Male | Reference | Reference | Reference |
| Female | 1.07 (0.95, 1.21) | **1.25 (1.06, 1.48)** | 1.21 (0.97, 1.51) |
| **Insurance** |  |  |  |
| Private only | Reference | Reference | Reference |
| Public only | **1.49 (1.27, 1.75)** | **1.93 (1.51, 2.46)** | **1.35 (1.02, 1.80)** |
| Public and private | 1.23 (0.89, 1.71) | **1.90 (1.19, 3.04)** | 0.67 (0.36, 1.23) |
| Other | **1.89 (1.28, 2.79)** | 1.58 (0.94, 2.65) | 0.93 (0.50, 1.72) |
| Uninsured | 1.09 (0.91, 1.31) | 1.09 (0.84, 1.41) | 1.18 (0.90, 1.56) |
| **CLS Exposure** |  |  |  |
| No | Reference | Reference | Reference |
| Yes | **4.01 (3.46, 4.65)** | **4.37 (3.48, 5.49)** | **1.41 (1.11, 1.78)** |
| *AAPI = American Asian and Pacific Islander, AIAN = American Indian and Alaskan Native, CLS = Criminal legal system, CUD = Cannabis Use Disorder*  *Note: Bolded values indicate p < 0.05* | | | |
